# Multiparametric Analysis of PET and Quantitative MRI for Identifying Intratumoral Habitats and Characterizing Trastuzumab-Induced Alterations

**DOI:** 10.3390/cancers17152422

**Published:** 2025-07-22

**Authors:** Ameer Mansur, Carlos Gallegos, Andrew Burns, Lily Watts, Seth Lee, Patrick Song, Yun Lu, Anna Sorace

**Affiliations:** 1 Department of Biomedical Engineering, University of Alabama at Birmingham, Birmingham, AL 35294, USA; 2 Spencer Fox Eccles School of Medicine, University of Utah, Salt Lake City, UT 84112, USA; 3 Department of Biomedical Engineering, University of Georgia, Athens, GA 30602, USA; 4 Department of Radiology, Duke University School of Medicine, Durham, NC 27710, USA; 5 Department of Radiology, University of Alabama at Birmingham, Birmingham, AL 35294, USA; 6 O’Neal Comprehensive Cancer Center, University of Alabama at Birmingham, Birmingham, AL 35294, USA

**Keywords:** multiparametric PET/MRI, HER2-positive breast cancer, imaging biomarkers, tumor heterogeneity

## Abstract

**Background/Objectives:** This study investigates the utility of multiparametric PET/MRI in delineating changes in physiologically distinct intratumoral habitats during trastuzumab-induced alterations in a preclinical HER2+ breast cancer model. **Methods:** By integrating diffusion-weighted MRI, dynamic contrast-enhanced MRI, [^18^F]Fluorodeoxyglucose- and [^18^F]Fluorothymidine-PET, voxel-wise parametric maps were generated capturing cellular density, vascularity, metabolism, and proliferation. BT-474 tumor-bearing mice have high expression of HER2 and, in response to trastuzumab, an anti-HER2 antibody, effectively show changes in proliferation and tumor microenvironment alterations that result in decreases in tumor volume through time. **Results:** Single imaging metrics and changes in metrics were incapable of identifying treatment-induced alterations early in the course of therapy (day 4) prior to changes in tumor volume. Hierarchical clustering identified five distinct tumor habitats, which enabled longitudinal assessment of early treatment response. Tumor habitats were defined based on imaging metrics related to biology and categorized as highly vascular (HV), hypoxic responding (HRSP), transitional zone (TZ), active tumor (ATMR) and responding (RSP). The HRSP cluster volume significantly decreased in trastuzumab-treated tumors compared to controls by day 4 (*p* = 0.015). The volume of ATMR cluster was significantly different at baseline between cohorts (*p* = 0.03). The TZ cluster, indicative of regions transitioning more to necrosis, significantly decreased in treated tumors (*p* = 0.031), suggesting regions had already transitioned. Multiparametric image clustering showed a significant positive linear correlation with histological multiparametric mapping, with R^2^ values of 0.56 (HRSP, *p* = 0.013, 0.64 (ATMR, *p* = 0.0055), and 0.49 (responding cluster, *p* = 0.024), confirming the biological relevance of imaging-derived clusters. **Conclusions:** These findings highlight the potential utility of multiparametric PET/MRI to capture biological alterations prior to any single imaging metric which has potential for better understanding longitudinal changes in biology, stratifying tumors based on those changes, optimizing therapeutic monitoring and advancing precision oncology.

## Simple Summary

This study explores how combined PET/MRI imaging can track early biological changes within HER2-positive breast tumors treated with trastuzumab, an anti-HER2 therapy. A preclinical mouse model and multiple imaging techniques were used to map tumor features like cell density, blood supply, metabolism, and proliferation. Assessing single imaging measures or their changes could not detect treatment effects early on (day 4). However, by clustering imaging data into five distinct “tumor habitats” with unique biological properties, early treatment responses were observed before tumor shrinkage. For example, the hypoxic responding cluster decreased significantly in treated tumors by day 4. The imaging-derived habitats matched well with tissue-based measurements, confirming their biological relevance. Overall, this approach may improve early assessment of treatment effectiveness, support tumor stratification, and advance personalized cancer therapy by capturing subtle tumor microenvironment alterations before structural changes appear.

## 1. Introduction

Intratumoral heterogeneity is known to contribute to tumor progression, as diverse genetic and phenotypic subregions create a complex microenvironment that enables the development of therapeutic resistance. Such heterogeneity imposes selective pressures within the tumor microenvironment, enabling certain subpopulations of cancer cells to survive in the presence of therapy [1]. Characterizing these variations can allow for improved prediction of therapeutic outcomes and for the development of more effective, personalized treatment strategies. HER2+ breast cancer is an aggressive subtype of breast cancer characterized by overexpression of the HER2 receptor, leading to rapid tumor progression and poor prognosis [2]. The combination of chemotherapies and targeted therapies, particularly trastuzumab, a monoclonal antibody that targets HER2, has improved patient outcomes. However, clinical responses remain suboptimal as fewer than 35% of patients initially respond to trastuzumab, and of those responders, approximately 70% experience disease progression within a year [3,4,5]. This highlights the critical need for improved characterization of trastuzumab-induced biological alterations to better tailor HER2-treatment approaches. Preclinical studies have identified several noninvasive imaging biomarkers to assess these changes in a HER2+ breast cancer model treated with trastuzumab. For example, greater decreases in [^18^F]Fluorothymidine positron emission tomography ([^18^F]FLT-PET) uptake compared to [^18^F]Fluorodeoxyglucose ([^18^F]FDG-PET) suggest that early on trastuzumab alters cellular proliferation [6,7,8,9]. Additionally, the use of [^18^F]FMISO-PET and multiparametric magnetic resonance imaging (MRI) has provided insights into trastuzumab-induced hypoxic and vascular alterations, respectively [10,11]. Given the extensive literature characterization of this model, it provides a robust framework to validate a novel multiparametric imaging and image processing pipeline.

Noninvasive imaging techniques can be utilized to characterize and monitor longitudinal biological and molecular changes prior to downstream alterations in tumor volume. While radiomic approaches are promising, they often lack a direct correlation between the extracted imaging features and underlying biological processes that drive tumor alterations. By integrating molecular and functional imaging data, noninvasive imaging modalities provide a biologically grounded representation of intratumoral heterogeneity, which is essential for advancing cancer treatment strategies. Quantitative MRI approaches, such as diffusion-weighted MRI (DW-MRI) and dynamic contrast-enhanced MRI (DCE-MRI), provide critical insights into tumor physiology. DW-MRI uses motion-sensitive gradients to capture changes in the Brownian motion of water molecules, yielding the apparent diffusion coefficient (ADC). This metric serves as a surrogate for cellular density—a parameter that has shown promise as an early biomarker of response to neoadjuvant chemotherapy in breast cancer [12]. DCE-MRI generates parametric maps of vascular permeability (*K^trans^*) and extracellular extravascular volume (*v_e_*) through mathematical fitting of rapidly acquired *T*_1_-weighted data during a contrast agent administration. DWI- and DCE-MRI have been shown to predict therapeutic response in both preclinical models and clinical studies of breast cancer [10,12,13,14,15]. Specifically, increases in ADC have been shown to be correlated with decreases in cellularity and responding tumors [12]. Vascularity metrics have been more complicated to assess, but when combined with proliferative and metabolic data can be used to differentiate vascularized aggressive tumors from aggressive tumors with hypoxic environments. The selection of these biomarkers was performed based on the extensive literature demonstrating their utility in HER2+ breast cancer models, particularly in evaluating response to trastuzumab. This multimodal imaging and image processing approach provides a comprehensive multiparametric view of tumor’s underlying biology.

This multiparametric imaging approach has an opportunity to noninvasively identify the heterogeneity and changes of complex tumor biology. Leaning on multiparametric MRI that has been shown to be predictive of therapy response in both preclinical and clinical breast cancer, and complementary molecular imaging techniques such as [^18^F]FDG-PET and [^18^F]FLT-PET can further enhance this multiparametric framework by assessing glucose metabolism and cellular proliferation, respectively [10,14,16,17]. In this study, we investigate the utility of voxel-based characterization of intratumoral habitats using combined molecular and functional biologically relevant habitats from PET/MRI in a preclinical HER2+ breast cancer model with histological validation. To explore this potential, we developed an acquisition and processing pipeline for preclinical imaging of solid tumors, which integrates data from separate PET and MRI scanners. This framework leverages the CT scans associated with the PET for attenuation correction and tumor registration to MRI. Once coregistered, PET and MRI datasets are combined via agglomerative hierarchical clustering to define intratumoral habitats, or regions of physiologically similar characteristics. This pipeline facilitates voxel-wise multiparametric mapping, enabling for the assessment of intratumoral heterogeneity and the longitudinal assessment of the biologically driven changes in response to targeted therapeutics. Clustering methods were applied to delineate distinct intratumoral habitats, which were then used to quantify longitudinal alterations following trastuzumab treatment. Histological validation was performed to correlate imaging-derived habitats with the underlying biological alterations, ultimately aiming to guide cancer treatment strategies by identifying nonresponding tumors early on during the course of therapy.

## 2. Materials and Methods

### 2.1. Animal Model and Treatment Response Assessment

Human-derived HER2+ breast cancer cells (BT-474) were utilized to develop a flank tumor model in mice, as outlined prior [7]. Once tumors reached ~250 mm^3^, mice were assigned to either a control group receiving saline or a treatment group receiving 10 mg/kg of trastuzumab (anti-HER2 targeted therapy) on days 0 and 3 (N = 8). Tumor response was assessed over the imaging time course using caliper measurements. A separate cohort was treated and monitored longitudinally to confirm long term response to treatment. All imaging protocols with preclinical MRI (9.4T Bruker BioSpec, Bruker, Billerica, MA, USA) and PET/CT (Sofie GNEXT, Sofie, Sterling, VA, USA) to evaluate tumor changes was performed on day 0 and repeated on day 4 using a 3D printed device for registration across platforms.

### 2.2. Magnetic Resonance Imaging

High resolution T_2_-weighted MRI was acquired to serve as a reference for tumor and tissue delineation using a 256 × 256 × Z, TR/TE = 2000 ms/24 ms, and a voxel resolution of 0.13 × 0.13 × 1 mm with Z referring to the number of slices ranging from seven to thirteen. Tumor and muscle tissue regions of interest (ROIs) were manually segmented on the *T*_2_-weighted-MRI in VivoQuant (Invicro, Needham, MA, USA).

Diffusion-weighted MRI was acquired with a standard pulse gradient spin echo sequence with four *b*-values (0, 150, 500, and 800 s/mm^2^). The slicing geometry was matched to the T_2_-weighted MRI with an acquisition matrix of 64 × 64 × Z, TR/TE = 4500/22.7 ms, flip angle = 90°, and a voxel size of 0.53 × 0.53 × 1 mm. Voxel-wise DWI signal intensity data was fit to Equation (1) in order to produce apparent diffusion coefficient maps.

(1) $$ADC=\frac{ln\left(\frac{S_{0}}{S\left(b\right)}\right)}{b}$$

*S*_0_ is equal to the signal intensity of the voxel at *b* = 0, *b* is the strength of the diffusion gradient, and *S*(*b*) is the signal intensity of the voxel at a given gradient strength. Slices of the DWI data with a mean above the median of the tumor + 2 standard deviations were excluded from the ADC analysis.

Dynamic Contrast Enhanced MRI requires the collection of pre-contrast T1 maps, followed by dynamic temporal imaging. Pre-contrast *T*_1_ relaxation maps were generated through the acquisition of variable repetition sequences with a flip angle of 180°, voxel spacing of 0.53 × 0.53 × 1 mm, TE of 10 ms, and a TR of 255, 400, 800, 1500, 3000, and 5500 ms. Voxel-wise fitting of the MRI signal acquired at each of these repetition times was performed in MATLAB 2023 following Equation (2).

(2) $$S=S_{0}*\left(1-e^{\left(-TR/T_{1}\right)}\right)$$

where *S* is the measured signal intensity at a specific repetition time TR. The longitudinal relaxation time T_1_ and the equilibrium signal intensity *S*_0_ were simultaneously estimated by fitting the equation to the measured signal intensities using MATLAB’s nonlinear least squares fit. Voxels with non-positive signal intensities post fitting or those which did not converge were assigned as invalid (NaN) in the final *T*_1_ maps.

The DCE-MRI acquisition consisted of rapid longitudinal *T*_1_-weighted sequences using a fast low angle shot sequence with a 64 × 64 × Z matrix, TR/TE = 100/1.65 ms, flip angle of 30°, and a temporal resolution of 6.4 s. Following twenty frames of pre-contrast acquisition, ProHance (279.3 mg/mL) (Bracco Diagnostics, Monroe Township, NJ, USA) was diluted at a ratio of 40 µL per mL of saline. A total volume of 200 µL of the diluted contrast agent was administered per subject via a tail vain catheter. Voxel-wise DCE data were fit to the Extended Tofts model presented in Equation (3) [18].

(3) $$C_{t}\left(t\right)=V_{p}C_{p}\left(t\right)+K^{trans}\int_{0}^{t}C_{p}\left(\tau \right)e^{-\left(\frac{K^{trans}}{V_{e}}\right)\left(t-\tau \right)}d\tau$$

The concentration of the contrast agent in the tissue at time *t*, derived from the voxel’s DCE signal intensity, is modeled as the sum of two components (1) the product of *V_p_*, the plasma volume fraction, and the population-derived arterial input function (*C_p_*(*t*)) [19,20]; and (2) the product of *K^trans^*, the volume transfer constant, and the convolution of the arterial input function (*C_p_*(*t*)) with an exponential decay function accounting for the contrast agent exchange between the plasma and volume of extracellular extravascular space (*v_e_*) [18]. The population-based arterial input function (AIF) was calibrated for each DCE scan using a segment of skeletal muscle tissue, serving as a reference [21], prior to modeling of pharmacokinetic parameters. Additionally, the AIF was resampled and matched temporally to correspond with the arrival of the contrast agent [10]. Voxels were excluded from further analysis if their parameter values exceeded physiologically relevant thresholds (*K^trans^*: 0–5 min^−1^; *v_e_* and *v_p_*: 0–1), or if their enhancement did not reach at least 25% increase from baseline. This exclusion accounted for 37.15 ± 14.21% of total voxels per tumor.

### 2.3. PET/CT Imaging

[^18^F]FDG and [^18^F]FLT PET/CT were performed after MRI acquisition. Mice were intravenously administered a dose of [^18^F]FDG (100 ± 10 µCi) in a total volume of 100 µL. 20 min static PET imaging was performed one-hour post-injection using the Sofie GNEXT PET/CT scanner. After imaging, the tracer was allowed to decay for a minimum of six half-lives, ensuring tracer washout and decay. Subsequently, the mice were administered a dose of [^18^F]FLT (100 ± 10 µCi) in a total volume of 100 µL and another 20 min static PET was acquired one-hour post-injection. A CT-based attenuation correction was applied to the PET images, followed by conversion to standard uptake values (SUV) via Equation (4).

(4) $$SUV=Activity/\frac{Injected\;Dose}{Body\;Weight}$$

### 2.4. Developing a Multimodal Registration Pipeline

**Figure 1:**
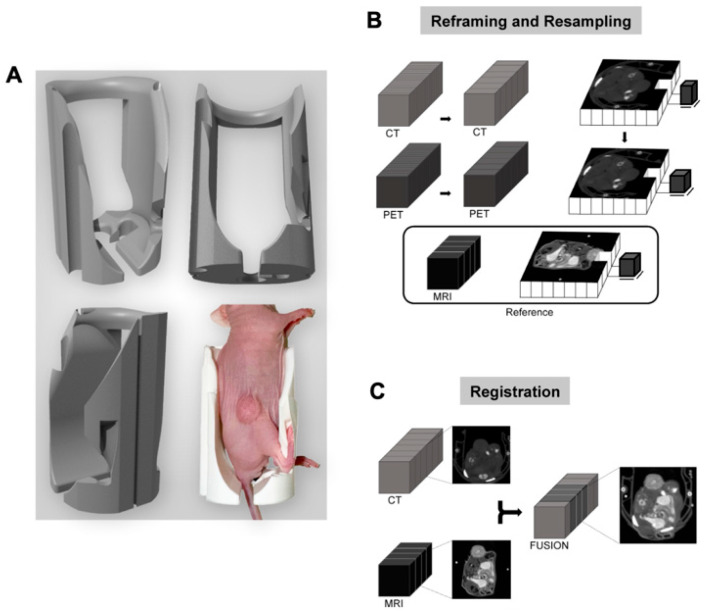
(**A**) A custom 3D-printed CT/MR compatible mouse couch used to ensure accurate scan alignment across imaging acquisitions. (**B**,**C**) Registration workflow for PET/CT and MRI, including reframing, resampling, and registration to align PET/CT images with the parametric MRI maps.

A custom 3D-printed mouse couch (Figure 1A) was utilized to establish a platform for scan registration across imaging modalities (3D-print file available upon request). After image acquisition and the generation of parametric maps, the PET/CT images were adjusted to match the lower resolution of the MRI parametric maps (Figure 1B,C). PET/CT images were, first, reframed by averaging adjacent slices to match the 1 mm slice thickness of the MR acquisitions. These reframed images were then linearly down sampled with anti-aliasing to match the voxel size of the parametric MR maps (0.53 × 0.53 mm^2^), ensuring that each voxel across modalities consistently represented the same physical space. Finally, the reframed and resampled PET/CT images were registered to the MR images using an affine 3D transform, optimized with a regular step gradient descent algorithm over 100 iterations. Mattes mutual information was used as the optimization metric, with increased weighting applied to a cropped tumor region to enhance alignment accuracy. Supplemental Figure S1 demonstrates the matched MRI, CT1 (associated with [^18^F]FDG), and CT2 (associated with [^18^F]FLT). Since all MRI sequences are inherently linked, the CT scans associated with each PET scan were first registered to the high-resolution *T*_2_-weighted MRI using the described method. The resulting transformation matrix was then applied to the PET images to achieve parametric map alignment. This registration workflow was performed using VivoQuant (Invicro).

### 2.5. Discovery of Intratumoral Imaging Habitats

A five-dimensional matrix was generated by pooling voxel-wise values of the sampling-matched ADC, *K^trans^*, *v_e_*, FDG-SUV, and FLT-SUV within the tumor ROI. Each feature was normalized to a mean of zero and standard deviation of one for equal contribution of each parameter. Multiparametric imaging data were then processed using voxel-wise hierarchical agglomerative clustering with five clusters, applied to the five-dimensional matrix derived from the registered imaging maps, adapted from Syed et al. [22]. To ensure physiological relevance, voxels were filtered based on predefined physiological ranges, outlined in Supplemental Table S1. This clustering approach facilitated the identification of distinct intratumoral subregions, or habitats, which represent physiologically meaningful variations within the tumor microenvironment. The optimal number of clusters (*k* = 5) was determined using the resulting dendrogram and mean silhouette score, selected from a range of two to eight clusters evaluated for the combined cohort. Characterization of the discovered intratumoral habitats was performed by analyzing the multidimensional centroids of each cluster and assigning a physiological relevant habitat to each cluster. These habitats were measured longitudinally between day 0 and day 4 and the changes in composition between control and treated were compared. A multiregional spatial interaction (MSI) analysis, adapted from Wu et al. [23], was implemented to examine the spatial organization of distinct subregions (clusters) within each tumor, specifically assessing spatial contiguousness. Following clustering of the multiparametric imaging data, the frequency of cluster-to-cluster border interactions in 3D space was quantified. These counts were normalized to cluster size to represent the fraction of all neighboring voxel pairs assigned to specific clusters. To determine whether certain subregions were spatially associated more or less than expected by chance, the observed tumor arrangement was compared to a randomized control, in which the overall size of each subregion was preserved while its spatial location was shuffled. This comparison allowed for the identification of non-random spatial patterns, identifying deviations from an expected homogeneous distribution. To further validate these spatial patterns, a z-score analysis was performed by comparing the real MSI values to a randomized spatial distribution with the same cluster volumes and distributions.

### 2.6. Immunohistochemical Staining and Imaging Processing

On day 4 post-imaging, mice were euthanized, and tumors were sectioned transversely at the largest cross-section to align with the axial imaging plane. Samples were paraffin-embedded, sectioned, and stained for H&E, Ki67, CD31, GLUT1, and pimonidazole to assess cellularity, proliferation, vascularity, glucose metabolism, and hypoxia, respectively [24]. Whole-slide images of the samples were acquired at 20× magnification (EVOS M7000 microscope, ThermoFisher, Waltham, MA, USA) and quantitatively analyzed for expression (QuPath v0.5). Briefly, living and necrotic tissue was semi-automatically annotated on H&E whole tissue samples, with necrotic regions excluded from further analysis (37.47 ± 13.31% necrotic area per tumor). Living tissue segmentation maps were generated and applied to all scans. H&E and Ki67 processing followed previously described methods [7], while a custom pipeline was developed for processing CD31, GLUT1, and pimonidazole-stained tissue into quantitative maps. For CD31-stained images, mean fluorescence intensity was used to classify samples as high or low vascularity, followed by dilation, smoothing, and hole-filling operations to refine vessel structures. GLUT1-stained images followed the same classification, but only cytoplasmic fluorescence signals were quantified. Pimonidazole-stained tissue was processed by identifying cells with a cytoplasmic DAB optical density of 0.7. Each processing step resulted in segmentation maps, which were then used to register all stains to the H&E reference for spatial alignment.

Processed histology maps were generated by quantifying positive and negative cell detections for each stain, followed by a whole tumor section registration using segmentation masks. These maps were then down sampled using a uniform spatial smoothing kernel of size 10 pixels and resampled with bilinear interpolation to closer match the lower resolution of the imaging and minimize clustering computation. Feature extraction was performed via this convolution-based density estimation and down sampling. Valid pixels, associated with living tissue, were identified, and feature data were aggregated across mice of both cohorts, and normalized feature-wise. Hierarchical agglomerative clustering using Ward’s linkage and Euclidean distance was applied to the combined feature dataset which allowed for classification of distinct histological clusters. Cellular density of clusters was assessed through nuclei count normalized to habitat area of H&E-stained sections. CD31-stained images were used to evaluate vessel count per habitat area to quantify vascularity. Ki67+ nuclei and GLUT1+ area, normalized to habitat area, were used as measures of cellular proliferation and glucose metabolism of each habitat, respectively. Cluster voxels were mapped back to individual tumors, which enabled for visualization and quantification of intratumoral variations.

Processing code is available at https://github.com/mansurlab/MutliParamPreclincialPETMR (accessed on 1 May 2025).

### 2.7. Correlating PET/MR Imaging Habitats with Histological Habitats

**Figure 2:**
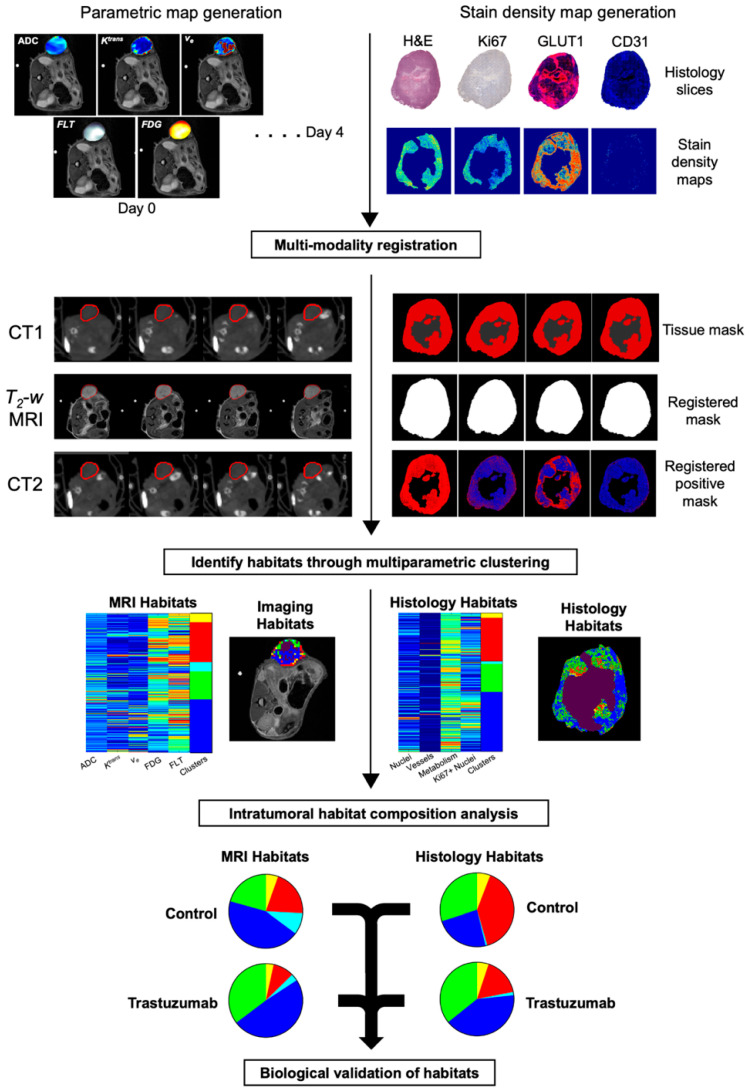
Study overview for multiparametric PET/MRI and histology-based tumor habitat analysis. Longitudinal PET and MRI data were acquired and processed to generate multiparametric maps of the tumor (left). Each tumor voxel is represented as a row vector, with each element corresponding to an imaging-derived quantitative parameters. Voxel data from all mice were pooled and clustered to identify distinct tumor habitats (third row, left). Tumor composition was then assessed over time by calculating the percentage of tumor volume occupied by each habitat (bottom row, left). For histological validation, end-point tissue samples were processed to generate stain density maps (top row, right). Each pixel was characterized by a row vector of stain density values, and pooled pixel data were clustered to define histology-derived tumor habitats (third row, right). Tumor composition was quantified as the percentage of tumor area occupied by each histological habitat (bottom row, right). Biological validation of the imaging-derived habitats was performed by correlating the percent tumor volume of imaging-derived habitats and the percent tumor area of histology-derived habitats.

To biologically validate the imaging clusters, correlations were performed between the imaging-derived habitats and histological habitats. The central slice was selected based on the highest correlation between imaging-derived habitat volume percentages and matching histology-derived habitat percentages within a five-slice window (the central slice ± 2 slices). This selected central slice was then used to compare the correlation between imaging-derived and histology-derived tumor habitat compositions. Percent tumor volume for each habitat in the selected central slice was correlated with the percent tumor area of the matching histological habitats. Figure 2 provides an overview of the study workflow for identifying and characterizing intratumoral multiparametric PET/MRI habitats. Longitudinal changes in these imaging-derived habitats were evaluated over time. A similar pipeline was applied to histological samples, and the final clustered maps were used for biological validation of the imaging-derived habitats.

## 3. Statistical Analysis

Significant differences between the mean parameter values between imaging-derived and histology-derived habitats were evaluated with a one-way analysis of variance (ANOVA) followed by Tukey’s Honest Significant Difference (HSD) test. Differences in habitat composition at baseline over time between treatment conditions were assessed using a non-parametric, two-tailed, Mann–Whitney Wilcoxon Test. Comparisons between control and treated groups were performed also using non-parametric, two-tailed, Mann–Whitney Wilcoxon Test. Correlations between imaging and histology-derived habitat percentages were evaluated using Pearson’s product-moment correlation. Two correlation analyses were conducted to first identify which histology-derived clusters best correlate to the imaging-derived clusters and second to assess the relationship between imaging-derived and histology-derived tumor habitats. The first analysis matched the clusters identified from histological data to imaging-derived clusters by systematically permuting cluster area percentages and comparing them to the corresponding tumor habitat volumes. The permutation yielding the highest correlation with tumor volumes was selected, and physiological labels were assigned accordingly. A *p*-value of less than 0.05 was considered statistically significant. Statistical significance is indicated as follows: *p* ≤ 0.05 (*), *p* ≤ 0.01 (**), *p* ≤ 0.001 (***).

## 4. Results

### 4.1. Early Treatment Induced Alterations Are Masked When Measured as Single Imaging Metric Changes

**Figure 3:**
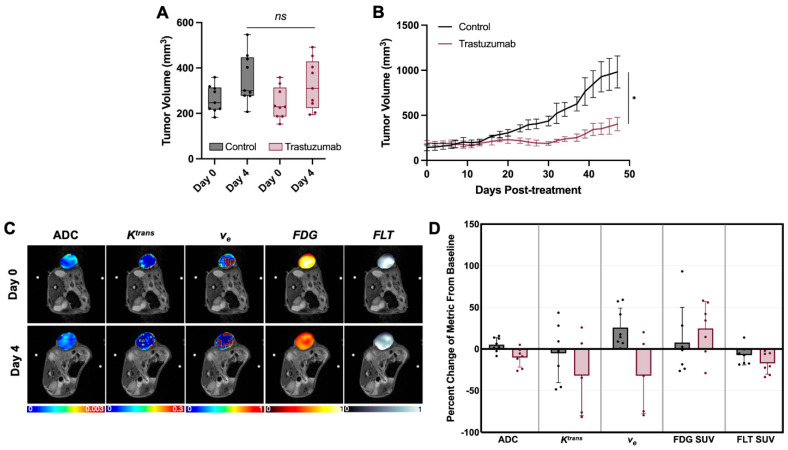
Tumor volume measurements (**A**) of control and trastuzumab-treated tumors at baseline and on day 4. Longitudinal tumor volume (**B**) trends over time. Representative parametric MRI and PET maps (ADC, *K^trans^*, *v_e_*, FDG-SUV, FLT-SUV) at baseline and day 4 (**C**). Percent change from baseline (**D**) of each imaging metrics comparing control and treated groups. *p* ≤ 0.05 (*).

No significant differences in tumor volume were observed between control and trastuzumab-treated tumors at baseline or on day 4, as shown in Figure 3A. As expected, a significant decrease in tumor volume (*p* = 0.035) was observed in the longitudinal cohort assessing downstream treatment response as demonstrated in Figure 3B. The absence of significant volume reduction at day 4 suggests that any biomarker changes observed at this early time point may serve as prognostic biomarkers. Representative parametric MRI and PET maps, including ADC, *K^trans^*, *v_e_*, FDG-SUV, and FLT-SUV images at baseline and day 4, are shown in Figure 3C. These images illustrate the various complementary data that can be derived from each modality to noninvasively characterize various aspects of tumor physiology over time. When evaluating the percent change from baseline for each imaging metric (Figure 3D), certain trends were observed, such as decreased ADC, *v_e_*, and FLT, but no significant differences in single metrics emerged between control and trastuzumab-treated tumors at this time point.

### 4.2. Discovery of PET and MRI Tumor Habitats

Following parametric map generation, multimodal images were registered to ensure spatial consistency. The custom 3D-printed mouse couch was the most effective tool for alignment across imaging modalities. Supplemental Figure S1 presents representative images of the aligned scans. Segmentations performed independently on sampled anatomical scans, were compared to assess tumor registration accuracy with Dice similarity coefficient calculated after registration. The Dice scores between MRI and CT1, MRI and CT2, and CT1 and CT2 were 0.95 ± 0.01, 0.92 ± 0.01, and 0.95 ± 0.02, respectively, indicating high tumor volume registration accuracy.

**Figure 4:**
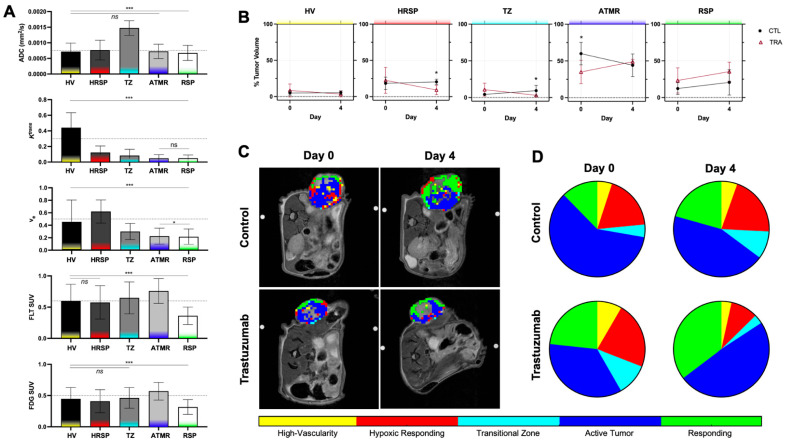
Cluster parameter values (**A**) plotted for each imaging metric. The dotted line represents the median value of that metric with intensity levels classified as high (above median) or low (below median) to further aid in cluster characterization. Statistically significant differences in mean parameter values across nearly all clusters. Longitudinal comparison of temporal changes in tumor composition characteristics (**B**) presented as percent of tumor volume including highly vascular (HV), hypoxic responding (HRSP), transitional zone (TZ), active tumor (ATMR), and responding (RSP). Representative baseline and day 4 images of the clustering results (**C**) remapped to the spatial domain for but control and treated mice. (**D**) Tumor composition analysis showing the relative volume of each cluster over time, highlighting dynamic changes in response to treatment and differences between control and treated tumors. Statistical significance is indicated as follows: *p* ≤ 0.05 (*), *p* ≤ 0.001 (***).

After registering and pooling the multiparametric data, clustering analysis identified five distinct clusters. Statistically significant differences were observed across nearly all clusters (*p* < 0.05), as shown in Figure 4A. This demonstrates that agglomerative hierarchical clustering is capable of distinguishing physiologically unique intratumoral subregions without spatial inputs. To further define the physiological relevance of each cluster, the mean parameter values across all metrics were compared on a cluster-wise basis longitudinally in Figure 4B. The median parameter values across all clusters were plotted for each metric, with intensity levels classified as high when above the median and low when below.

Figure 4C presents representative images mapping the spatial distribution of clusters in control and treated tumors at baseline and on day 4. The first cluster, highlighted in yellow, exhibited a mean ADC of 7 × 10^−4^, along with *K^trans^* and *v_e_* values of 0.44 and 0.45, respectively. These parameter values indicate a relatively low ADC, suggesting a dense cellular environment [25], while the high *K^trans^* and *v_e_* reflect increased vascularity and a larger extracellular and extravascular space. Given its moderate proliferation and metabolism, this habitat was termed as high vascularity (HV). The red cluster was characterized by low *K^trans^*, high *v_e_*, and low FDG uptake, indicative of regions responding to treatment. The combination of high extracellular space and low vascularity and low metabolic activity suggests that while these areas exhibit reduced perfusion, they appear hypoxic yet responsive, leading to their classification as hypoxic responding (HRSP). At baseline, the HRSP cluster volumes were comparable (*p* = 0.84); however, following two treatment administrations, this cluster exhibited an average 59.19% reduction in its percentage of the tumor volume, compared to a 10.72% increase in controls (Figure 4B). By day 4, the HRSP volume in treated tumors was significantly lower than in controls (*p* = 0.015). Notably, the HRSP (red) cluster volume decreased from 22.15% to 9.19% of the tumor volume in treated tumors (*p* = 0.095), while insignificant this trend was not observed in controls (*p* = 0.69). The third cluster, defined by the highest ADC values (above 1 × 10^−3^) with median values for other parameters, suggests regions transitioning (TZ) toward necrosis. Treatment significantly decreased this cluster volume (*p* = 0.031). The dark blue cluster, which constituted the majority of the tumor, was characterized by high [^18^F]FLT and [^18^F]FDG but low *K^trans^*, indicative of less vascularity and therefore more hypoxic, aggressive active tumor regions (ATMR). Significant volume changes in this cluster were observed between control and treated tumors at baseline (*p* = 0.03), and in treated tumors the volume trended towards increasing (*p* = 0.15). However, by day 4, no significant difference was observed, as ATMR volume decreased in controls but increased in treated tumors, it suggests a shift in tumor composition. The final cluster, represented in green, exhibited low values across all measured parameters and was therefore classified as a responding region (RSP) through decreases in cellular activity. While an overall increase in RSP was observed in both groups, with a greater overall presence in treated tumors compared to controls (*p* = 0.30 vs. *p* = 0.54). Tumor composition by cluster volume is visually depicted in Figure 4D.

### 4.3. Discovery of Histological Tumor Habitats

**Figure 5:**
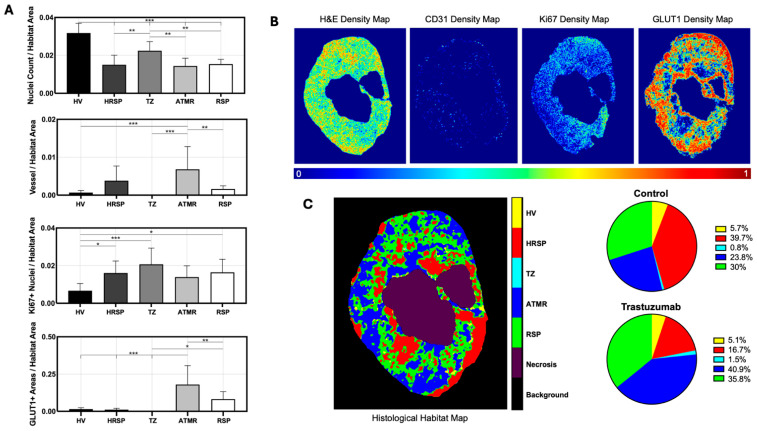
(**A**) Physiological characterization of histology-derived clusters using nuclei count (H&E) for cellular density, vessel count (CD31) for vascularity, Ki67+ nuclei for proliferation, and GLUT1+ area for glucose metabolism, all normalized to habitat area. (**B**) Representative stain density maps for H&E, CD31, Ki67, and GLUT1. (**C**) Spatially remapped histology-derived tumor habitats, visualized as multiparametric histology maps. Analysis of tumor composition revealed increased ATMR (blue) and RSP (green) habitats in treated tumors, with a corresponding decrease in the HRSP (red) cluster, aligning with imaging findings from day 4. *p* ≤ 0.05 (*), *p* ≤ 0.01 (**), *p* ≤ 0.001 (***).

To physiologically characterize the clusters derived from agglomerative hierarchical clustering of histological staining data, the mean parameter counts, or area was compared across clusters (Figure 5A). 5B presents normalized representative stain density maps for H&E, CD31, Ki67, and GLUT1. Following clustering and characterization, voxels were remapped to their spatial locations, generating multiparametric histology-based habitat maps, as shown in Figure 5C. Analysis of tumor composition revealed an increase of 72.17% and 19.27%, respectively, in ATMR (blue) and RSP (green) habitats in treated tumors compared to controls, closely mirroring the imaging results from day 4. Additionally, a 136.13% decrease in the HRSP (red) cluster was observed in the treated group, suggesting a treatment-induced decrease in the percentage of hypoxic responding regions within the tumor composition. Validation of hypoxic regions present in the ATMR (blue), RSP (green), and HRSP (red) clusters was performed by identifying colocalization of the cluster map with the pimonidazole positive stain density map. The distribution of total hypoxia did not vary between ATMR and HRSP 43.51 ± 21.91% of total hypoxia vs. 29.91 ± 10.67% (*p* = 0.09). Meanwhile, RSP had significantly lower hypoxia compared to the ATMR at 16.87 ± 10.86% of total hypoxia (*p* = 0.02).

To assess spatial heterogeneity, MSI analysis was conducted to evaluate the spatial distribution and contiguousness of the identified clusters (Supplemental Figure S2). The fraction of neighboring voxel pairs was plotted against cluster identity to examine spatial relationships. Cluster 4 (ATMR) displayed the highest self-pairing across time points and treatment conditions, indicating that this cluster remained spatially homogeneous and was frequently contiguous with itself. Additionally, the HRSP, ATMR, RSP which accounted for a large proportion of the tumor volume, showed high internal cluster contiguousness, as evidenced by a predominance of diagonal bars in the MSI plot. Positive z-score differences between the tumor MSI and the randomized MSI were observed across all diagonal boxes, with a mean z-score of 22.96, indicating that, on average, these values were 22.96 standard deviations above the mean of the randomized distribution. Thus, confirming that the identified clusters were spatially coherent rather than randomly dispersed within the tumor microenvironment.

### 4.4. Positive Linear Correlations Between Imaging-Derived and Histology-Derived Habitats

**Figure 6:**
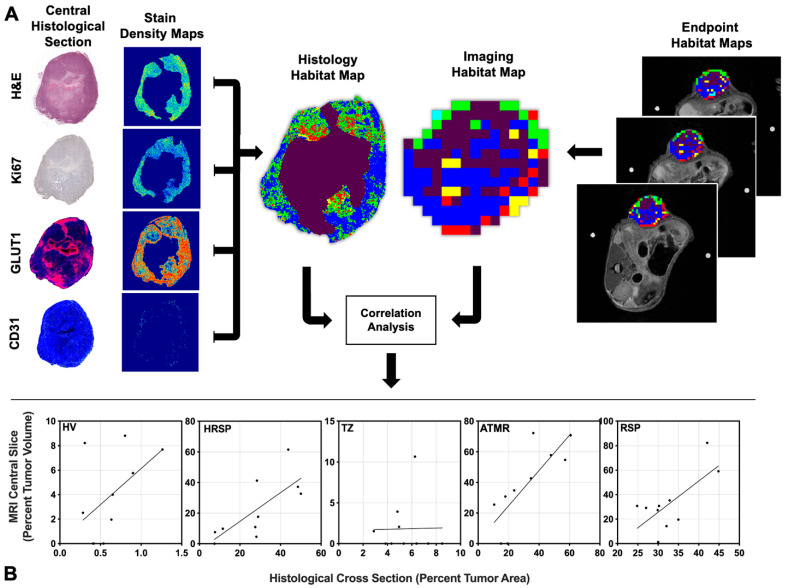
Schematic representation of the correlation workflow (**A**), where histology-derived clusters were matched to imaging-derived clusters based on optimized permutations of cluster area and tumor habitat volume. Scatter plots depicting significant positive linear correlations (**B**) between the percent tumor volume of Clusters 2, 4, and 5 in the central MRI slice and the corresponding percent tumor area in histological cross-sections (R^2^ = 0.559, 0.6397, and 0.491, respectively; *p* = 0.0129, 0.0055, and 0.024).

Figure 6A, examined the correlation between the percent tumor volume of each cluster in the central MRI slice and the percent tumor area of the corresponding histological cross-section. Significant positive linear correlations were observed for Clusters 2, 4, and 5, (corresponding to HRSP, ATMR, and RSP) which together accounted for more than 80% of the tumor composition (R^2^ = 0.559, 0.6397, and 0.491, respectively; *p* = 0.0129, 0.0055, and 0.024) as shown in Figure 6B. These findings support a strong spatial concordance between imaging-derived and histology-derived habitats, biologically validating the parametric clustering approach.

## 5. Discussion

Intratumoral heterogeneity is a major driver of tumor progression, which creates selective pressures that enable the survival and proliferation of diverse intratumoral subpopulations [1]. Detecting treatment-induced biological alterations prior to measurable tumor volume changes is critical for understanding therapeutic response. This study therefore utilized multiparametric PET/MRI to spatially resolve distinct intratumoral habitats in a preclinical HER2+ breast cancer model, integrating imaging parameters from clinically- and biologically relevant quantitative imaging approaches (DCE-MRI, DW-MRI, and [^18^F]FDG- and [^18^F]FLT-PET). This HER2+ breast cancer model was selected due to its well-documented alterations in these imaging biomarkers and its distinct separation of long-term response to trastuzumab [6,7,8,11,22]. Conventional quantitative and semiquantitative imaging techniques often reduce tumor heterogeneity to a single metric, losing critical spatial and temporal information. In contrast, multiparametric imaging integrates multiple biological properties assessed through different modalities, which enables a more comprehensive assessment of both the tumor and its microenvironment.

In this study, parametric maps from MRI and PET imaging (ADC, *K^trans^*, *v_e_*, FDG-, and FLT-PET) were co-registered. Multi-dimensional voxel data across all time points and treatment conditions were aggregated and analyzed using hierarchical agglomerative clustering. Prior to tumor characterization, necrotic regions, defined as voxels with less than 25% enhancement on DCE-MRI, were excluded from further analysis, as the goal of the study was to characterize living tissue. The observed necrotic volume of 37% was expected [14] and aligned with the necrotic area observed on the central tumor slice histology. Combining data across groups and timepoints allows for the capturing of all potential biological clusters rather than just the ones that present at a singular time point. This approach also eliminates the assumption that all habitats are present in all tumors, ensuring consistent habitat definitions over time. This methodology allowed for a quantitative assessment of trastuzumab-induced alterations in intratumoral habitat volume changes compared to control.

Without explicitly providing spatial data, five distinct tumor habitats were identified based on imaging parameters alone, reflecting underlying differences in cellular density, vascularity, extracellular extravascular space, metabolism, and cellular proliferation. Based on their multi-dimensional imaging profiles, these habitats were classified as high vascularity (HV), hypoxic responding (HRSP), transitional zone (TZ), active tumor (ATMR) and responding (RSP) (4A). At baseline, all cluster volumes were comparable between control and treated tumors, except for the active tumor cluster. Over time, this cluster’s volume decreased in control tumors, whereas in treated tumors, it increased, in both cases stabilizing at to approximately 46% of the tumor on day 4, encompassing nearly half of the tumor volume in both conditions. These findings suggest that multiparametric imaging could help identify baseline tumor signatures predictive of response to therapies with variable efficacy, such as immunotherapy and radiation therapy. Further characterization of the active tumor cluster revealed high metabolic and proliferative activity, along with low extracellular extravascular space, indicative of an aggressive phenotype typical in growing cancers. Longitudinal imaging demonstrated that while tumor growth slowed significantly, these tumors continued to expand over time, suggesting that certain subpopulations remain unaffected by therapy (Figure 3B). This observation aligns with prior studies evaluating proliferation as a biomarker of response in this model [6,8]. For example, some studies report a decrease in FLT uptake, SUV_max_ of the tumor, after one week of therapy, while others note a reduction in proliferation as early as a single therapy administration [6,8]. This variation in timing suggests that intratumoral proliferation is dynamic, and requires baseline or longitudinal imaging to accurately assess treatment response. As expected, the high vascularity cluster, characterized as well-perfused regions near vascular structures, accounted for less than 10% of tumor volume across all time points and conditions [14]. This minimal yet consistent vascularity aligns with the characteristics of cancerous, sporadic, non-normalized leaky vasculature. The hypoxic responding cluster exhibited elevated *v_e_* and low *K^trans^*, indicating that this subregion was had cells of decreasing growth, with high extracellular extravascular space and low vascularity, suggesting a hypoxic environment. This cluster’s volume significantly decreased following trastuzumab treatment, suggesting effective early modulation of hypoxia. The corresponding histological analysis supported this, indicating 29.91 ± 10.67% percent of the total pimonidazole staining within this cluster. These findings are consistent with previous studies evaluating trastuzumab’s capacity to reduce hypoxia, as evident by [^18^F]FMISO-PET and pimonidazole validation [11]. While hypoxia was not directly measured in this study, several multiparametric MRI studies have demonstrated a strong relationship between DCE-MRI parameters and hypoxia [10,13,26]. The responding cluster was distinguished by low values across all imaging parameters, representing a potentially quiescent or less active tumor region. Extending multiparametric imaging over a longer period is expected to increase the RSP cluster volume in trastuzumab-treated tumors, reflecting the later changes observed in studies evaluating single imaging metrics.

Multiparametric assessment of matched histological stained samples revealed a significant positive linear correlation among the three primary tumor clusters. The percent tumor volume of clusters in the central MRI slice was correlated with the percent tumor area in the corresponding histological cross-section. Furthermore, imaging-derived habitats exhibited higher spatial contiguity compared to randomized spatial distributions, as confirmed through MSI analysis (Supplemental Figure S2). The biological validity and reproducibility of the clusters are supported by (1) significant inter-cluster differences in imaging centroids (Results, Figure 4A) and (2) strong concordance with histology derived habitats (Results, Figure 6B). These findings support the identified tumor habitats and demonstrates that this multiparametric imaging and image processing approach effectively delineates physiologically distinct subregions, mapping them cohesively within the tumor and its microenvironment. The high contiguousness found in the ATMR cluster suggests that it was minimally impacted by therapy. The MSI analysis suggests that HRSP and RSP clusters were found in heterogenous pockets. While the imaging and histological tissues were matched, spatial registration between the imaging and tissues was not performed due to shrinkage and morphological alterations in the ex vivo samples following fixation and staining. As shown in Figure 5B, aligning histology-derived and imaging-derived clusters spatially for regional comparisons could provide additional noise due to mismatch of registration and therefore habitat volumes were assessed, providing global comparisons of biologically relevant subregions in the tumor. A key limitation of multiparametric preclinical PET/MRI is the spatial resolution constraints of the imaging modalities, limited both by physics and temporal/spatial acquisition tradeoffs for in vivo imaging. These limitations can lead to partial-volume underestimations of tracer uptake, especially in small or poorly delineated subregions. To mitigate this issue, CT-based attenuation correction was applied to PET data, improving border uptake accuracy. Next, all PET parametric maps were linearly down sampled with anti-aliasing to match the lowest spatial resolution scan (DCE-MRI). Although these steps reduce much of the bias, a sight underestimation of SUV in small habitats may persist. Future work should investigate the implementation of relative uptake measurements and partial-volume correction algorithms to further enhance quantitative uptake accuracy and subregion delination [27]. Cluster classification in this study was performed using manual thresholding, where high and low values were determined using median-based cutoffs. While this approach provided an effective initial characterization, future studies will implement physiologically defined ranges to improve reproducibility across imaging modalities. Importantly, this HER2+ breast cancer model was chosen due to its well-characterized imaging biomarker changes and established response to trastuzumab, providing a biologically validated framework for model development. This foundation enables the application of this approach to other tumor models, facilitating the exploration of biologically relevant tumor habitats and their response to various therapies. As more imaging parameters are incorporated into multiparametric clustering, the interpretability of results becomes increasingly complex. Integrating multiple biomarkers enhances biological characterization; however, distinguishing meaningful patterns from redundant or noisy data remains challenging. To address this, dimensionality reduction techniques were applied, leading to the exclusion of metrics such as *v_p_* from DCE-MRI parameter mapping.

The potential clinical utility of multiparametric PET/MRI lies in its ability to integrate physiological imaging biomarkers to generate composite metrics that improve tumor characterization. This approach has demonstrated significant clinical value in multiple cancer types. For example, in gliomas, multiparametric PET/MRI has been used to quantify treatment response and estimate pharmacokinetic parameters predictive of progression-free survival [28]. In prostate cancer, the addition of [^68^Ga]GaPSMA-PET to multiparametric MRI improved sensitivity while maintaining specificity, compared to multiparametric MRI alone [29]. In breast cancer, combining mean transit time (DCE-MRI) and ADC with radiomic features from [^18^F]FDG-PET and ADC significantly improved the ability to differentiate between benign and malignant lesions (AUC = 0.983) [30]. Given this demonstrated ability to distinguish tumor phenotypes based on noninvasive imaging-assessed underlying biology, the same principles can be applied to treatment response paradigms in patient data by leveraging multiparametric PET/MRI or PET + MRI data. This preclinical dataset combined clinically relevant quantitative approaches that have been integrated into oncology trials and in standard-of-care approaches. Cluster analysis using a subsection of imaging approaches could also be considered by combining high-resolution vascular and cellular assessments from MRI with any functional information from PET within a single imaging session to identify early physiological alterations indicative of therapeutic efficacy. As more therapies target distinct pathways and combination treatments are becoming increasingly common, multiparametric imaging may enable a deeper understanding of intratumoral biological changes that biopsies alone cannot capture. Additionally, ongoing efforts in the standardization of quantitative parametric mapping techniques across multiple vendors has improved reproducibility and clinical translation [31]. These applications highlight the growing role of multiparametric imaging in integrating multiple biological dimensions, enhancing tumor characterization, and advancing personalized cancer care. The findings of this study further support the potential of multiparametric PET/MRI in identifying physiologically distinct tumor habitats, which could improve response assessment and treatment planning in HER2+ breast cancer.

## 6. Conclusions

This study demonstrates the potential of multiparametric PET/MRI for spatially resolving distinct intratumoral habitats and characterizing trastuzumab-induced alterations in a preclinical HER2+ breast cancer model. By integrating functional and molecular imaging modalities—including DW-MRI, DCE-MRI, and [^18^F]FDG- and [^18^F]FLT-PET—this approach overcomes the limitations of single-metric tumor assessments, preserving critical spatial and temporal heterogeneity. The identification of five physiologically distinct tumor habitats highlights the capability of multiparametric imaging to capture complex biological variations, providing insights into cellular density, vascularity, metabolism, and proliferation. Longitudinal tracking of these habitats revealed dynamic treatment responses, with changes in hypoxic responding and active tumor subregions, further validated through histological correlation. These findings demonstrate the potential of multiparametric imaging not only for preclinical research but also for clinical translation, where improved characterization of tumor subpopulations through imaging could enhance response prediction and therapeutic decision-making in HER2+ breast cancer.

## Data Availability

Scripts to process data and example data can be found at: https://github.com/mansurlab/MutliParamPreclincialPETMR (accessed on 1 May 2025).

## Supplementary Materials

The following supporting information can be downloaded at: https://www.mdpi.com/article/10.3390/cancers17152422/s1, Figure S1: Presents representative images of the aligned scans; Figure S2: MSI plots comparing cluster spatial contiguousness; Table S1: Defined physiological bounds of imaging metrics.
