# Supplementary material for: Multiparametric Analysis of PET and Quantitative MRI for Identifying Intratumoral Habitats and Characterizing Trastuzumab-Induced Alterations
Source: Cancers (Basel). 2025 Jul 22;17(15):2422. doi: 10.3390/cancers17152422 (PMC12345643; doi:10.3390/cancers17152422)
Supplement: Supplementary file 1 [file cancers-17-02422-s001.zip › cancers-3706758-supplementary.pdf]

Supplemental Materials:

Supplemental Table S1. Defined physiological bounds of imaging metrics.

Imaging Metric	Lower Bound	Upper Bound
ADC	0.0001	0.002
K^{trans}	0.001	1
v_e	0.001	1
qT1	[0.001	10000
[^{18}F]FDG	0.001	5
[^{18}F]FLT	0.001	5

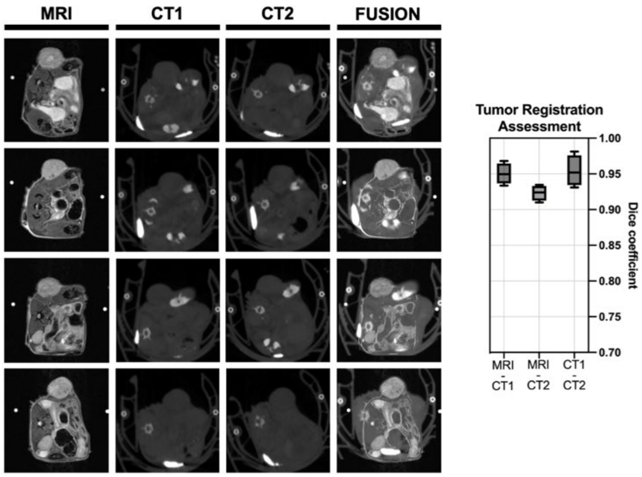

Supplemental Figure S1. Presents representative images of the aligned scans. CT1 corresponds to the CT associated with FDG scan. CT2 corresponds to the CT associated with FLT scan. Dice scores are presented post-registration of the tumor volume segmented on each scan separately.

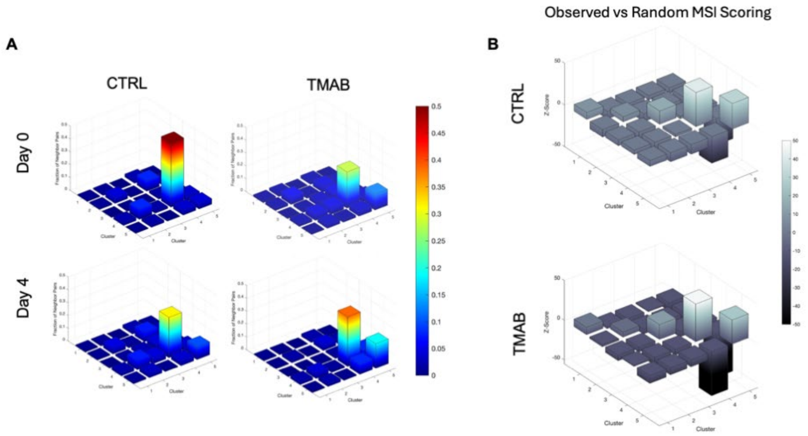

Supplemental Figure S2. MSI plots comparing cluster spatial contiguousness. (A) demonstrates the fraction of neighboring pairs across clusters under various conditions. (B) A z-score analysis was conducted by comparing the observed MSI values to a randomized spatial distribution with identical cluster volumes and distributions. Positive z-score differences were observed in all diagonal boxes confirming that the identified clusters were spatially coherent rather than randomly dispersed within the tumor microenvironment.
